# Fruit Waste as a Matrix of Health-Promoting Compounds in the Production of Corn Snacks

**DOI:** 10.1155/2022/7341118

**Published:** 2022-03-03

**Authors:** Dorota Gumul, Rafal Ziobro, Marek Kruczek, Justyna Rosicka-Kaczmarek

**Affiliations:** ^1^Faculty of Food Technology, Department of Carbohydrate Technology and Cereal Processing, University of Agriculture in Krakow, 31-120 Krakow, al. Mickiewicza 21, Poland; ^2^Lodz University of Technology, Faculty of Biotechnology and Food Sciences, Institute of Food Technology and Analysis, 90-537 ul Stefanowskiego 2/22 Łódź, Poland

## Abstract

Extrusion is an industrial technology allowing utilization of fruit-based off-products, rich in bioactive and prohealth compounds, in the production of gluten-free snacks. The use of up to 20% fruit waste (apple waste) in the production of such snacks results in significant increases of polyphenols and flavonoids, as well as individual phenolic acids: chlorogenic (36 times) and cryptochlorogenic (4 times). UPLC-PDA-MS/MS method allowed to observe huge increases in phloridzin (25 times), epicatechin (8 times), catechin (up to 6 times), and the end procyanidin (almost 3 times) in corn snacks. The most suitable addition level was 20% because it resulted in the highest increases in the abovementioned compounds and dietary fiber, which provided high antioxidant potential of corn-apple snacks. Therefore, the production of such snacks could be recommended on industrial scale as they have the best organoleptic properties.

## 1. Introduction

According to the data published by many authors, about 12% of apple off-products in Poland is directed to landfill sites to the detriment of the environment and economy, while the rest is mostly used as a raw material for the production of soil conditioning compost, pectin, or as an additive to animal feed [1, 2]. Apple waste can also be used as a raw material for the production of biogas, ethyl alcohol, and organic acids (for example, citric acid could be obtained by using *Aspergillus niger*) and finally could be sold out as fiber preparations [1–3].

Taking into account the practical aspects of apple pomace utilization presented above, it should be clearly stated that the prohealthy character of this heterogeneous plant mixture is taken into account in a small part. According to Kennedy et al. [4] and Carson et al. [5], dried apple pomace is composed of seeds, stalks, and flesh with skin and core, in quantities 2.6–4.2%, 0.5–1.1%, and 84.1–95.7%, respectively. This variability of morphological parts in the pomace can guarantee that after drying, i.e., when microbiologically stable, it can become a potential source of many valuable compounds, especially dietary fiber and phyto-compounds including polyphenols [6]. Thus, they constitute a specific food matrix being a source of valuable prohealth compounds, dietary fiber (35-65 g/100 g d.m.), and especially bioactive compounds classified as polyphenols (262-856 mg/100 g d.m.) [6]. Physiological activities of the abovementioned compounds include hypoglycemic, hypocholesterolemic, anticancer effects, reduction of postprandial glycemia and hypertension, antiallergic, antibacterial, anticoagulant, anti-inflammatory, antiviral effects, and reduction of the risk of diseases such as atherosclerosis and other cardiovascular diseases, bone degenerative changes, cataracts, diabetes, genetic damage, and neurodegenerative diseases, including Alzheimer's disease [6–8]. Moreover, these compounds bind heavy metal ions and bile acids, improve intestinal peristalsis, have prebiotic properties, and decrease the risk of obesity [6–8].

Apple pomace as a matrix of prohealth compounds could be effectively utilized in one of the most popular processing technologies in modern food industry—extrusion. Such processing allows obtaining of new, innovative products with the addition of otherwise unacceptable raw materials, even regarded as wastes [9], in the current case—apple pomace. In this way, the waste management in fruit processing could be simplified, and the new product could be enriched in dietary fiber and phenolic compounds, which are abundant in apple pomace. An important element is also the introduction of a new gluten-free product, especially in the context of the increasing number of people suffering from celiac disease, whose diet is often based on products low in nutrients, fiber, and health promoting ingredients. The study on corn extrudates enriched in apple pomace seems fully justified in this context.

The aim of the study was to evaluate the influence of varying levels of apple waste (from 5 to 20%) on the content of dietary fiber and also contents of total polyphenols, flavonoids, phenolic acids, flavonols, flavon-3-ols, and dihydrochalcones in corn snacks. This evaluation was accompanied by the assessment of antioxidant potential of the corn snacks produced by means of a twin-screw extruder using 3 different methods (DPPH, ABTS, and FOMO methods).

## 2. Materials and Methods

### 2.1. Materials

Study material consisted of apple pomace and corn snacks supplemented with apple pomace at levels 5, 10, 15, and 20%.

The formulations used for snack production (5%, 10%, 15%, 20%) were composed of the ingredients presented in Table 1. Prior to extrusion, the pomace and corn grits were milled to granulation 750-1250 *μ*m, thoroughly mixed and conditioned to receive moisture level 14% ± 0.5%. Extrusion was performed in a corotating twin screw extruder Fudex (type 2FS60, Italy) at 100 rpm. Screw diameter equaled 60 mm, and temperatures of individual sections were as follows: 130°C, 160°C, and 125°C. The nozzle contained two openings with a diameter 3 mm.

The level of 20% apple pomace addition was the maximum used to produce the snacks, above which there occurred a rapid deterioration in the expansion, texture, and organoleptic characteristics of the snacks due to the excessive amount of dietary fiber in the sample.

### 2.2. Methods

Antioxidant constituents and antioxidant activities were determined in the ethanol extracts. 0.6 g of the sample was dissolved in 30 mL 80 g/100 g ethanol, shaken in a darkness for 120 min (electric shaker: type WB22, Memmert, Schwabach, Germany), and centrifuged (15 min, 4500 rpm. 1050 × g) in a centrifuge type MPW-350 (MPW MED. Instruments, Warsaw, Poland). The supernatant was decanted and stored at -20°C for further analyses.

Determination of total polyphenol content (TPC) was done by spectrophotometric method using Folin-Ciocalteu reagent (with F-C reagent), according to Singleton et al. [10].

5 cm^3^ of the extract was taken and diluted to 50 cm^3^ with distilled water. From the solution prepared in this way, 5 cm^3^ of the diluted extract was taken; 0.25 cm^3^ of Folin-Ciocalteu reagent (previously diluted with distilled water 1 : 1 *v*/*v*) and 0.5 cm^3^ of 7% Na_2_CO_3_ were added. The mixture was then stirred on a Vortex (type WF2, from Janke and Kunkel, Staufen, Germany) and left for 30 minutes in the dark. After this time, the absorbance was measured in a spectrophotometer (Helios Gamma, 100-240, Runcorn, England) at *λ* = 760 nm. The results were converted into mg of catechin/100 g d.m. or mg of gallic acid/100 g d.m.

The content of flavonoids was evaluated using a spectrophotometric method, according to El Hariri et al. [11]. 0.5 cm^3^ of ethanol extract was taken into a test tube, and 1.8 cm^3^ of distilled water and 0.2 cm^3^ of 2-aminoethyldiphenylborate reagent were added. The contents of the tube were mixed on a Vortex (type WF2, by Janke and Kunkel, Staufen, Germany), and the absorbance was measured in a spectrophotometer (Helios Gamma, 100-240, Runcorn, England) at *λ* = 404 nm. The flavonoid content was expressed as mg of rutin/100 g d.m.

#### 2.2.1. Determination of Individual Polyphenols by UPLC-PDA-MS/MS

*(1) Extraction*. Samples (1 g) were extracted with 10 mL of a mixture containing methanol of a purity level of HPLC (30 mL/100 mL), ascorbic acid (2.0 g/100 mL), and acetic acid in the amount of 1.0 mL/100 mL of the reagent. Extraction was carried out twice by incubation for 20 min under sonication (Sonic 6D, Polsonic, Warsaw, Poland) and repetitive (4 times) mixing. The suspension was then centrifuged at 19000 g for 10 min, and the supernatant was filtered through a 0.20 *μ*m Hydrophilic PTFE membrane (marble filter Sampility Millex, Merck, Darmstadt, Germany) and used for analysis.

*(2) Assay*. Phenolic compounds were analyzed using Aquity Ultra-Performance liquid chromatograph equipped with Binary Solvent Manager (BSM), Sample Manager (SM) combined with a PDA detector, and quadrilateral time of flight (Q-TOF) (Waters, Manchester, United Kingdom). The analysis was carried out on a 2.1 × 100 mm UPLC BEH C18 column containing 1.7 *μ*m particles (Waters, Manchester, United Kingdom). The selection of isocratic elution of the gradient was chosen as the elution mode in which it was applied as follows: 2 g/100 mL formic acid (A) and acetonitrile (B) in water as a mobile phase at a speed of 0.45 mL/min. Elution was started at 99 g/100 mL A for one min; after 12 min, the linear gradient was applied up to 75 g/100 mL B. The temperature of the column was 30°C, and the injection volume was 5 *μ*L. The working parameters of the mass detector were as follows: 2.5 kV capillary voltage and 30 V conical sample voltage. Ion source temperatures and desolvation were 130°C and 350°C, respectively. Nitrogen was used as a carrier gas at a flow rate of 300 L/h. The analyses were conducted in the full scan mode in the range of 100-1500 m/z, with a tolerance of 0.001 Da and a resolution of 5000. Internal reference standards, leucine and enkephalin, were continuously introduced through the lock-spray reference channel. Chromatograms were analyzed using a base peak (BPI) calibrated to 12,400 cps (100%). Data was collected and analyzed using the MassLynx v 4.1 (Waters) software. Anthocyanins were analyzed in the positive ion mode and the remaining polyphenols in the negative ion mode. Their identification was carried out by comparing the spectra of maximum UV absorption, molecular weight defined as mass/charge ratio, and retention times, as well as fragmentation spectra with available literature data. The spectra of degradation were obtained as a result of collision-induced dissociation (CID) in tandem mode. Collision energy was selected individually for each of the analyzed substances. The characteristic UV spectra were collected at the following wavelengths: *λ* = 320, phenolic acids; *λ* = 360, flavonols; *λ* = 280, flavan-3-ols; and *λ* = 340, flavones. Quantification of phenolic compounds was performed using external standard curves, using reference compounds selected on the basis of the target analyte/structure standard (chemical structure or functional group). The calibration curve for p-coumaric acid was used for the quantitative determination of 3-O-p-p-coumaroylquinic acid. Chlorogenic, cryptochlorogenic, and neochlorogenic acids were determined quantitatively according to own standard. (+) Catechin, (-) epicatechin, and procyanidin B2 were quantified according to own standard. Calibration curves for quercetin-3-O-rutinoside, 3-O-glucoside, and 3-O-galactoside were used for quantitative determination of quercetin derivatives. For the quantitative determination of isorhamnetin, isorhamnetin 3-O-rutinoside and 3-O-glucoside were used. All determinations were carried out in three repetitions (*n* = 3). The standards were prepared in concentrations ranging from 0.05 to 5 mg/mL. The correlation coefficient was *R*^2^ ≤ 0.9998. The results were expressed in mg per 100 g d.m [12].

Antiradical activity was assessed using analytical methods with DPPH [13] and by the formation of phosphomolybdenum complex, according to Prieto et al. [14]. Additionally, antiradical activity was assessed using analytical methods with ABTS (2,2′-azino-bis(3-ethylobenzothiazoline-6-sulphonic acid)-diammonium salt) [15]. The abovementioned methods were performed using a spectrophotometer. Results of antiradical activity were expressed as TEAC (Trolox equivalent antioxidant capacity-mg Trolox/g dry mass of sample).

#### 2.2.2. Organoleptic Analysis

The enriched corn snacks were evaluated by a group of 15 panelists with checked sensory sensibility. The analyses were performed in a lab designed and equipped according to PN-ISO-8589 (1998) [16]. Quality attributes are as follows: shape and appearance (weighting 0.2), consistency (weighting 0.25), structure and texture (0.15), and taste and smell (0.4) were evaluated using 5-point scale; 1 point, unacceptable; 2 points, poor; 3 points, acceptable; 4 points, desirable; and 5, highly desirable.

#### 2.2.3. Determination of Dietary Fiber

Content of total dietary fiber and soluble and insoluble fraction of dietary fiber was assessed by methods 32-07 AACCI (2012) [17] that is approved methods of the American Association of Cereal Chemists International, 1st ed. St Paul, MN: American Association of Cereal Chemists.

#### 2.2.4. Statistical Evaluation of Results

All analyses were performed at least in duplicate, and the obtained results were subjected to analysis of variance (ANOVA) using the Statistica 13 statistical software package (StatSoft, TIBCO Software Inc.). The significance of differences between the mean values was verified by Duncan's test at *α* ≤ 0.05. Pearson correlation coefficient (MS Excel) was calculated additionally.

## 3. Results and Discussion

### 3.1. Apple Waste as a Source of Health-Promoting Compounds

The content of polyphenols in apple pomace was 919.2 mg of catechin/100 g d.m. (Table 2).

In the study of Ćetković et al. [18], total polyphenols varied from 420 to 867 mg of chlorogenic acid/100 g d.m., and according to Sudha et al. [19], their content equaled 551 mg of gallic acid/100 g d.m. Persic et al. [20] determined the content of total polyphenols in a range 19-50 mg of gallic acid/100 g d.m. On the other hand, Leyva-Corral et al. [21] reported the level of 324 mg of gallic acid/100 g d.m. Li et al. [22] determined the content of polyphenols in apple pomace at the level 556 mg of gallic acid/100 g d.m. Leon et al. [23] estimated the content of polyphenols in apple pomace to be 327.60 mg of gallic acid/100 g d.m. The content of polyphenols in plant material depends not only on extraction procedure but also on the expression of the results (e.g., different phenolic compounds used to calculate the amount of polyphenols) [6]. Therefore, to compare the results concerning total polyphenols with the reports of other authors, they were recalculated and expressed in mg of gallic acid/100 g d.m., which resulted in 469.5 mg of gallic acid/100 g d.m. Taking this into account, it could be stated that total content of polyphenols in analyzed apple pomace was comparable to earlier reports, and the observed differences were most probably due to the type and method of extraction.

Total content of flavonoids was determined as 277.62 mg of rutin/100 g d.m. (Table 2). According to Ćetković et al. [18], the level of flavonoids in apple pomace ranges between 45 and 119 mg of rutin/100 g d.m. According to Leon et al. [23], the content of flavonoids in apple pomace equals 75.6 mg of rutin/100 g d.m.

Apart from total content of polyphenols and flavonoids obtained by spectrophotometric methods, UPLC-PDA-MS/MS analysis of individual phenolic compounds present in apple pomace was also performed (Table 3).

Total content of phenolics determined by this method equaled 163.64 mg/100 g d.m. in apple pomace (Table 3). In the studies of Sato et al. [24] and Kołodziejczyk et al. [25], the content of polyphenols in apple pomace ranged between 262 and 856 mg/100 g d.m., while Kammerer et al. [26] reported 11.76 mg/100 g d.m., Leyva-Corral et al. [21] identified polyphenols on the level 114.54 mg/100 g d.m., and in the study of Ćetković et al. [18], their sum equaled 69.2-147.4 mg/100 g d.m.

Taking into account the contents of individual phenolic acids, the largest quantity was observed in the case of chlorogenic acid (22.62 mg/100 g d.m., Table 3), which is in general agreement with the results of other authors, who determined 92-104 mg/100 g d.m. [27]. In the study of Rabetafika et al. [28], Leyva-Corral et al. [21], Kammerer et al. [26], and Ćetković et al. [18], the quantities of this acid in apple pomace were as follows: 3.3-7.9 mg/100 g d.m., 41.55 mg/100 g d.m., 1.43 mg/100 g d.m., and 3-17.6 mg/100 g d.m. Liu et al. [29] determined the content of chlorogenic acid in apple pomace on the level 3.4 mg/100 g d.m.

Quercetin derivatives were abundant among flavonols (Table 3), and the major compounds were identified as quercetin-3-O-galactoside, 31.86 mg/100 g d.m.; quercetin-3-O-rhamnoside, 27.65 mg/100 g d.m.; and quercetin-3-O-xyloside, 20.76 mg/100 g d.m. Quercetin derivatives were also identified in apple pomace by other authors, who reported quercetin-3-O-glucoside level as 28.6-61.0 mg/100 g d.m. [18] and 52.1-68.1 mg/100 g d.m. [28], while in the current study, its concentration was 8.40 mg/100 g d.m. (Table 3). Liu et al. [29] determined the content of quercetin-3-O-galactoside, quercetin-3-O-rhamnoside, and quercetin-3-O-glucoside on the levels 10.45, 4.3, and 3.77 mg/100 g d.m, respectively.

Flavan-3-ols and dihydrochalcones are other groups of very important phenolic compounds present in apple pomace. Flavan-3-ols include as follows: catechin, 1.62 mg/100 g d.m.; procyanidin B2, 3.77 mg/100 g d.m.; and epicatechin, 1.32 mg/100 g d.m. (Table 3). Other researchers [18, 26, 28] determined the levels of catechin in apple pomace as follows: 1.7-12.7 mg/100 g d.m., 0.24 mg/100 g d.m., and 0.94-1.4 mg/100 g d.m., respectively. The amounts of epicatechin in the same raw material were found as 2.4-17.3 mg/100 g d.m., 0.93 mg/100 g d.m., 14-19 mg/100 g d.m., and 12.23 mg/100 g d.m. [18, 21, 26, 28]. The quantities of procyanidin B2 were 2.3-10 mg/100 g d.m., 0.93 mg/100 g d.m., and 9.3-16 mg/100 g d.m. [26–28]. Phloridzin is a major dihydrochalcone of apple pomace, as its level reaches 21.2 mg/100 g d.m. (Table 3). According to Leyva-Corral et al. [21], its quantity is about 17.97 mg/100 g d.m. and according to Ćetković et al. [18] in the range 0.7-8.5 mg/100 g d.m., while in the study of Kammerer et al. [26], its level was reported as 4.04 mg/100 g d.m. Wojdyło et al. [30] measured the concentration of phloridzin between 0.30 and 30.33 mg/100 g d.m. According to Liu et al. [29], the content of phloridzin was 6.85 mg/100 g d.m.

According to many sources [18, 22, 26, 28, 29], chlorogenic acid, phloridzin, quercetin-3-O galactosidase, quercetin-3-O xyloside, and quercetin-3-O rhamnoside are the main polyphenols in apple pomace.

The content of the abovementioned phenolic compounds depends on many factors, including the apple variety, climatic and soil conditions, agrotechnical conditions, production technology, and the way pomace samples are prepared for analysis [20, 31]. Hence, some discrepancies between the presented results and those of other authors may arise.

High quantity of phenolic compounds described above results in high antioxidant potential of apple pomace (Table 2), which was evaluated by three different methods. Two of these methods are aimed at determining antiradical activity (with the application of free radicals DPPH and ABTS) and the third to antioxidant activity (phosphomolybdenic method).

The amount of total dietary fiber was 64.2 g/100 g, with soluble fraction 13.47 g/100 g and insoluble fraction 50.74 g/100 g (Table 3). In the study by Waldbauer et al. [6], the amount of dietary fiber ranged from 35 to 65 g/100 g d.m., and Lyu et al. [32] showed a fiber content of 26.5 g/100 g in apple pomace. Dietary fiber is the major component of the dry matter of apple pomace [6]. Apple pomace contains significant amounts of the soluble fraction of dietary fiber, mainly pectin, which, among other things, binds heavy metal ions, has prebiotic properties, and lowers blood cholesterol and postprandial glycaemia [33]. Apple pomace is also a significant source of water-insoluble cellulose, which improves intestinal peristalsis and therefore reduces the risk of carcinogenesis and postprandial glycaemia [34].

Lignin, which belongs to the insoluble fraction of dietary fiber, binds bile acids and lowers cholesterol levels. In addition, the dietary fiber components of apple pomace have a beneficial effect on the prevention and treatment of overweight, cardiovascular disease, diabetes, and cancer of the gastrointestinal tract [35]. Our results are in agreement with the earlier ones of Waldbauer et al. [6] that more than two-thirds of apple fiber is insoluble and the proportion between soluble and insoluble fraction is approximately 1 : 2. Such a ratio is beneficial in terms of prohealth properties of the fiber. It should be kept in mind that fiber reveals hypocholesterolemic, hypoglycemic, and anticancerogenic properties [1, 2].

It should be emphasized that an integral part of fiber is the phenolic compounds discussed above.

It could be concluded that apple pomace is a matrix with high health-promoting potential and can therefore become an important tool in improving the health-promoting quality of many products, especially gluten-free ones.

### 3.2. Corn Snacks Enriched with Apple Pomace as a Matrix of Health Promoting Compounds and Their Organoleptic Analysis

Addition of apple pomace to snack formulation significantly increased the level of total polyphenols in extruded products. The increase ranged between 27% and 170% as compared to control and was parallel to the addition level of this postproduction material. Corn snacks with 5% apple pomace (sample EI 5% AP) contained 27% more flavonoids in comparison to control, and when the level of apple pomace was 20% (sample EI 20% AP), the increase of these bioactive compounds was higher by additional 143 percent points (Table 4). The share of apple pomace at the level of 10% and 15% in corn extrudates resulted in an increase in flavonoid content at a similar level compared to the control (on average by about 80%; Table 4).

At the same time, it can be suggested that although many authors claim that the extrusion process can cause losses of both polyphenols and flavonoids due to their degradation or polymerization with other compounds (by which their extractability is lower) [36] as well as phenolic acids by their decarboxylation, other reports indicate [37, 38] that proper setting of extrusion parameters (low moisture, high temperature) may cause increase of polyphenols through their release from cell walls of the extruded material. Equally important is the right type and amount of additive, which can provide an increase in the amount of these valuable bioactive components in the extrudates with their participation, despite the loss of phenolic compounds during this baro-thermal process [39–42]. In the study of Gumul et al. [40], concerning corn extrudates with a share of defatted blackcurrant seeds 2- to 10-fold increase in total polyphenols and 63%-186% rise in flavonoids could be observed in comparison to control. Reis et al. [43] observed that total content of polyphenols in extrudates with apple pomace (10%-30%) increased in a range 84%-168%, and the same change in anthocyanins was between 40% and 86% as compared to control. Extrudates with a 10% and 20% share of apple pomace were characterized by a threefold increase in flavonoid content, while 30% share of pomace caused a fourfold increase in the content of these bioactive compounds in relation to control [43]. According to the abovementioned authors [43], the observed increase in concentration of phenolic compounds in snacks is due to the presence of fruit pomace, which agrees with the presented results. Also, Preethi et al. [44] studying cereal-based extrudates with a share of apple pomace (5-25%) observed an increase in polyphenol and flavonoid contents which corresponded to the addition of apple pomace to the product. Cereal-based extrudates with a share of apple pomace contained from 4 to 16 times more polyphenols compared to the control. The addition of apple pomace to these extrudates resulted in a 2-fold to 12-fold increase in flavonoids relative to control. Leon et al. [23] also confirmed an increase in polyphenols in extruded products with yellow corn supplemented with apple pomace powder and a significant increase in flavonoids ranging from 15 to 200% relative to the control.

Analyzing the phenolic profile of corn extrudates with a share of apple pomace by UPLC-PDA-MS/MS method, it could be observed that even the control obtained from corn grits (sample EI CONTROL) contains high level of phenolic acids, which are partially endogenous acids originating from corn, namely, caffeoylglycerol, p-coumaroylquinic acid, 2-O-p-coumaroylglycerol, and feruloylquinic acid, which due to the applied processing parameters are freed from the cell walls of the cereal, which is why their level in the sample is high.

On the other hand, the extrudates with apple waste were characterized by a high content of chlorogenic and cryptochlorogenic acid, as a result of their introduction with apple waste, while the amount of these phenolic acids increased in parallel to the applied level of waste participation (Table 5).

Additionally, this significant increase in phenolic acids (chlorogenic even 36 times and cryptochlorogenic even 4 times) in lignocellulosic matrix of apple pomace could be generated by the combined action of temperature, pressure, and shear (especially high at low moisture), which cause disintegration of cell walls present in pomace during extrusion and facilitate the extraction of polyphenols from plant material thus influencing the results. The amount of remaining phenolic acids in the extrudate with apple pomace is related to the use of corn meal, because their amount decreases when corn meal is partially replaced with apple pomace. In the case of quercetin derivatives, it could be noticed that the content of these compounds in the extrudates is parallel to the applied pomace level. Corn snacks with a share of apple pomace were characterized by larger levels of catechin (even 6 times in sample EI 20% AP as compared to the control), procyanidin (even 3 times in sample EI 20% AP, as compared to control), and epicatechin (even 8 times in sample EI 20% AP in comparison to the control; Table 5). It could be explained by the fact that the oligomeric forms of these compounds present in apple pomace in the remnants of cell walls are depolymerized upon extrusion (high temperature, pressure, and shear) to dimers and monomers, which are more extractable, so their level is apparently increased. The application of apple pomace results also in an increase of the content of dihydrochalcones, mainly phloridzin in the extrudates (even 25 times more in comparison to the control; Table 5), which is a unique compound typical only for apple pomace. In the study of Liu et al. [29] concerning the extrudates with apple pomace, there was a twofold increase in chlorogenic acid and 44% rise in the content of epicatechin and dihydrochalcones.

High antioxidant potential of corn snacks with a share of apple pomace (Table 4) determined using three applied methods was highly correlated with the content of phenolic compounds detected in the products, which could be seen in the values of correlation coefficients between TPC and ABTS (*R*^2^ = 0.96), TPC and DPPH (*R*^2^ = 0.97), and TPC and phosphomolybdenic (*R*^2^ = 0.96). Additionally, the amounts of quercetin derivatives were highly correlated with ABTS (*R*^2^ = 0.98) and DPPH (*R*^2^ = 0.93) and the level of chlorogenic acid with ABTS (*R*^2^ = 0.99) and DPPH (*R*^2^ = 0.92), because correlation coefficients were especially high. Analyzing the abovementioned antioxidant potential, it should be stated that its values were mainly the result of the content of polyphenols in analyzed raw material; however, it could be possible that new compounds with antioxidant properties formed during extrusion, e.g., products of Maillard reaction or more active forms of polyphenols, could also influence the final results [23, 45]. The range of increase of antioxidant potential after the introduction of apple pomace to extrudates was from a few percent to three times in comparison to control (Table 4). Leon et al. [23] observed that when 30% apple pomace was added to extrudates, their antioxidant activity increased by 33% compared to control. And in the case of Preethi et al. [44], the increase in activity was 3.2-fold when using 5% apple pomace and even 13.5-fold when using 25% apple pomace in extrudates compared to the control.

**Figure 1:**
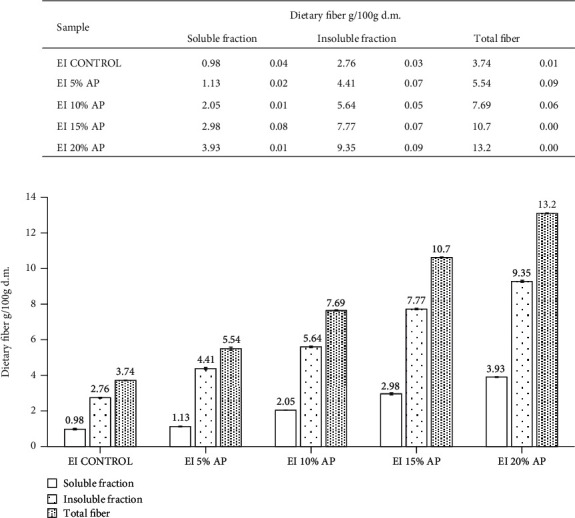
Dietary fiber in enriched corn snacks with apple pomace (for abbreviations, see Table 1).

Apple pomace is a valuable source of insoluble fractions of dietary fiber (50.74 g/100 g d.m., Table 3). It was found that together with increasing share of apple pomace, the level of these fiber fractions significantly increased (by 146% on average) in comparison to control. In the case of the extrudates with 5% share of apple pomace, the increase was 60%, and for those with 20%, almost 2.5-fold rise could be observed. Corn snacks with the highest share of apple pomace were characterized with the largest levels of insoluble fiber fractions (9.35 g/100 g d.m.) (Figure 1).

The content of the soluble fraction of dietary fiber in corn extrudates with apple pomace was related, similarly to the insoluble fraction, to the increasing proportion of apple pomace. An increase ranging from 15% to 301%, in the soluble fraction of dietary fiber, was observed in relation to the control in the extrudates with a share of apple pomace, with the highest increase achieved at the highest proportion (20%) of apple pomace (Figure 1).

This increase in the insoluble fraction of dietary fiber, however, was somewhat less than might be suggested by the level of apple pomace contribution. In the case of the soluble fraction, on the other hand, the recorded increase was above the expected value. This can be explained by the fact that the intensive barothermic processing during extrusion results in a partial breakdown of nonstarch polysaccharides and the formation of oligosaccharides. Additionally, both covalent and noncovalent bonds in carbohydrates are broken, which leads to their disintegration and formation of shorter and more soluble fragments of these carbohydrates and, consequently, conversion of insoluble fiber into its soluble fractions [46, 47].

The largest amount of total fiber could be found in the sample with the highest—20% share of apple pomace (13.28 g/100 g d.m.; Figure 1). Similar results were earlier obtained by Gumul et al. [48], in the studies on extrudates with a share of black currant seeds, in which the level of total fiber increased in the range 126%-660% in comparison to control, and Potter et al. [41], who observed 16-223% increase in dietary fiber in comparison to control when the fruit mix was applied in snack production. Ainsworth et al. [49] observed an increase in the level of dietary fiber ranging from 108% to 325% in comparison to control, when growing levels of barley malt were used for snack production. In contrast, Jozinovic et al. [50] in a study on apple pomace extrudates observed a decrease in insoluble dietary fiber of about 30% and an increase in soluble dietary fiber (in the range of 20-50%) and a decrease in total dietary fiber of about 15% with respect to the control. In a study by Tadesse et al. [51] on a sorghum-based extruded product supplemented with defatted soy flour (addition levels 10 and 20%), the total dietary fiber content increased by 52% and 86%, respectively, relative to the control. An analogous increase in total dietary fiber was shown in a study by Ruiz-Armenta et al. [52] on corn snack using the industrial by-products (bagasse) of naranjita fruit (*Citrus mitis* B.).

**Figure 2:**
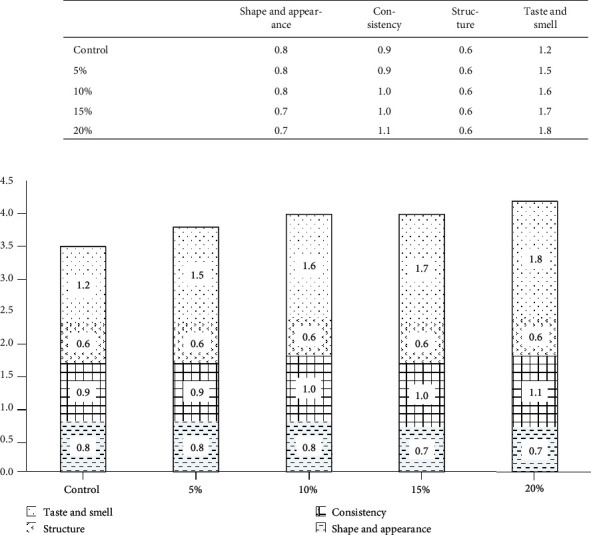
Organoleptic evaluation of enriched corn snacks (Control: control extrudate; 5%: extrudate with a share of 5% apple pomace; 10%: extrudate with a share of 10% apple pomace; 15%: extrudate with a share of 15% apple pomace; 20%: extrudate with a share of 20% apple pomace).

Organoleptic assessment of gluten-free extrudates with varying share of apple pomace proved that with an increasing share of pomace the overall acceptance of final product was improved (from 8% to 18% in comparison to control), mainly due to better taste and smell, and also ameliorated consistency. The best organoleptic scores were found for the extrudates with 20% share of apple pomace, as the abovementioned addition caused the best taste, smell, and consistency. No changes were detected in terms of structure and texture of extrudates with increasing share of apple pomace; despite in instrumental analysis, some increase of hardness could be observed, and high correlation between instrumental texture of starchy snacks and its organoleptic assessment was reported by Dehghan-Shoar et al. [53]. Most probably the changes in texture of extrudates were not considered by the panelists as important. The extrudates with 5% and 10% share of apple pomace as well as the control were characterized by similar shape and appearance, and the increase of the addition level to 15% and 20% caused a slight deterioration of these parameters (Figure 2).

Summarizing, apple pomace is a rich source of prohealth constituents, especially dietary fiber and polyphenols, which could be successfully used in the manufacture of gluten-free products. Our earlier studies [12] were focused on gluten-free bread with 5% addition of apple pomace, because it is known that bread plays a key role in supplying large quantities of nutritionally important compounds. In this work, another gluten-free product is described—corn snacks with 20% apple pomace as it is easy to produce on industrial scale and could be preferred by some consumers adhering to gluten-free diet. Expanding the portfolio of gluten-free products containing substantial levels of fiber and polyphenols seems to be the most important achievement of this study.

## 4. Conclusions

1. To sum up, the study confirmed that apple pomace is a matrix with high health-promoting potential and can therefore become an important tool in improving the health-promoting quality of many cereal products, especially gluten-free ones such as corn snacks
2. The apple pomace provides polyphenols and flavonoids, chlorogenic acid up to 36 times, cryptochlorogenic acid up to 4 times, catechin (up to 6 times) procyanidin (up to 3 times), and epicatechin (up to 8 times) in enriched corn snacks. The apple pomace also contributes to the increase of dihydrochalcones, mainly phloridzin in extrudates (up to 25 times in comparison to the control), which is a unique compound characteristic only for apple pomace
3. The addition of 20% of apple pomace contributes to the highest rise in the abovementioned bioactive compounds from the polyphenol group in corn extrudates, which translates into a high antioxidant potential of corn snacks with apple pomace. The extrudate with 20% of apple pomace contained higher levels of total, soluble, and insoluble dietary fiber, approximately 3 times more in comparison to control. Therefore, such an addition can be recommended for the production of innovative gluten-free snacks based on corn and apple pomace on an industrial scale, especially as its organoleptic scores were the best

## Figures and Tables

**Table 1:** Composition of formulations used for snack extrusion.

Sample	Corn grits (g)	Apple pomace (AP) (g)
EI CONTROL∗	5000	0
EI 5% AP	4750	250
EI 10% AP	4500	500
EI 15% AP	4250	750
EI 20% AP	4000	1000

∗AP: apple pomace; EI CONTROL: control extrudate (100% corn grits); EI 5% AP: extrudate with a share of 5% apple pomace; EI 10% AP: extrudate with a share of 10% apple pomace; EI 15% AP: extrudate with a share of 15% apple pomace; EI 20% AP: extrudate with a share of 20% apple pomace.

**Table 2:** Content of bioactive compounds from the polyphenol group in apple pomace and their antioxidant activities.

Phenolic compounds and antioxidant activities	Apple waste (apple pomace (AP))
Total polyphenols (mg of catechin/100 g d.m.)	919.20 ± 4.00
Total polyphenols (mg of gallic acid/100 g d.m)	469.5 ± 1.54
Flavonoids (mg of rutin/100 g d.m.)	277.62 ± 0.57
ABTS (mg Trolox/g d.m.)	12.16 ± 0.17
DPPH (mg Trolox/g d.m.)	3.09 ± 0.04
Phosphomolybdenic (mg Trolox/g d.m.)	27.69 ± 0.35

∗Different letters in columns denote mean values statistically different from each other.

**(a):** 

Flavonols^∗^							
Quercetin-O-rutinoside	Quercetin-3-O-galactoside	Quercetin-3-O-glucoside	Quercetin-3-O-arabinoside	Quercetin-3-O-xyloside	Quercetin-3-O-rhamnoside	Isorhamnetin-3-O-galactoside	Isorhamnetin-3-O-glucoside
3.80 ± 0.17	31.86 ± 0.21	8.40 ± 0.05	12.55 ± 0.03	20.76 ± 0.00	27.65 ± 0.00	1.30 ± 0.42	0.90 ± 0.00

**(b):** 

Phenolic acids^∗∗^			Flavan-3-ols			Dihydrochalcones	
Chlorogenic acid	Cryptochlorogenic acid	p-Coumaroyl quinic acid	(+) Catechin	Procyanidin B2	(-) Epicatechin	Floretin-2-O-xylo-glucoside	Floretin 2-O-glucoside (phloridzin)
22.62 ± 0.23	1.19 ± 0.07	1.83 ± 0.20	1.62 ± 0.03	3.77 ± 0.00	1.32 ± 0.23	1.92 ± 0.11	21.20 ± 0.08

**(c):** 

Dietary fiber		
Insoluble	Soluble	Total
50.74 ± 0.00	13.47 ± 0.05	64.21 ± 0.86

^∗^Luteolin 6-C-hexoside O-hexoside and luteolin O-hexoside C-hexoside were not detected. ^∗∗^Caffeoyl-dihydroxyphenyl-lactoyl-tartaric acid, 2-O-p-coumaroylglycerol, 1-O-p-coumaroylglycerol, p-coumaroylspermidine, di-p-coumaroylspermidine, feruloylquinic acid, and caffeoylglycerol were not detected.

**Table 4:** Total polyphenols and flavonoids in gluten-free extrudates enriched with apple pomace and antioxidant potential.

Sample	Total polyphenols (mg catechin/100 g d.m.)	Flavonoids (mg rutin/100 g d.m.)	ABTS method (mg Trolox/g d.m.)	DPPH method (mg Trolox/g d.m.)	FOMO method (mg Trolox/g d.m.)
EI CONTROL^∗^	65.68 ± 8.09 a^∗∗^	27.26 ± 0.66 a	1.93 ± 0.36 a	1.60 ± 0.03 a	23.72 ± 0.15 a
EI 5% AP	83.16 ± 4.66 b	34.71 ± 1.99 b	3.60 ± 0.06 b	1.69 ± 0.02 b	31.04 ± 0.38 b
EI 10% AP	114.36 ± 5.26 c	45.76 ± 0.66 c	5.75 ± 0.40 c	1.88 ± 0.01 c	39.30 ± 0.15 c
EI 15% AP	133.86 ± 4.65 d	51.92 ± 6.61 c	6.45 ± 0.13 d	2.07 ± 0.01 d	44.90 ± 0.15 d
EI 20% AP	177.58 ± 9.12 e	66.34 ± 0.66 d	7.46 ± 0.34 e	2.70 ± 0.00 e	48.56 ± 0.23 e

^∗^For abbreviations, see Table 1. ^∗∗^Different letters in columns denote mean values statistically different from each other.

**Table 5:** Quantitative and qualitative profiles of polyphenols in gluten-free extrudates enriched with apple pomace (mg/100 g d.m.).

Compounds	EI CONTROL^∗^	EI 5% AP	EI 10% AP	EI 15% AP	EI 20% AP
Luteolin 6-C-hexoside O-hexoside	0.00 ± 0.00 a^∗∗^	0.00 ± 0.00 a	0.00 ± 0.00 a	0.00 ± 0.00 a	0.00 ± 0.00 a
Luteolin O-hexoside C-hexoside	0.00 ± 0.00 a	0.00 ± 0.00 a	0.00 ± 0.00 a	0.00 ± 0.00 a	0.00 ± 0.00 a
Quercetin-O-rutinoside	0.00 ± 0.00 a	0.13 ± 0.03 b	0.37 ± 0.00 c	0.41 ± 0.05 c	0.47 ± 0.07 cd
Quercetin-3-O-galactoside	0.21 ± 0.00 a	1.38 ± 0.00 b	2.64 ± 0.09 c	3.85 ± 0.00 d	4.88 ± 0.00 e
Quercetin-3-O-glucoside	0.03 ± 0.00 a	0.34 ± 0.10 b	0.64 ± 0.00 c	0.91 ± 0.02 d	1.20 ± 0.05 e
Quercetin-3-O-arabinoside	0.08 ± 0.00 a	0.45 ± 0.08 b	0.81 ± 0.34 b	1.24 ± 0.03 c	1.53 ± 0.00 d
Quercetin-3-O-xyloside	0.10 ± 0.00 a	0.72 ± 0.21 b	1.27 ± 0.01 c	1.95 ± 0.00 d	2.50 ± 0.07 e
Quercetin-3-O-rhamnoside	0.08 ± 0.00 a	0.84 ± 0.03 b	1.60 ± 0.02 c	2.75 ± 0.04 d	3.63 ± 0.02 e
Isorhamnetin-3-O-galactoside	0.00 ± 0.00 a	0.07 ± 0.00 b	0.05 ± 0.00 b	0.06 ± 0.01 b	0.10 ± 0.03 b
Isorhamnetin-3-O-glucoside	0.00 ± 0.00 a	0.04 ± 0.00 b	0.12 ± 0.00 c	0.13 ± 0.00 c	0.20 ± 0.00 d
Chlorogenic acid	0.18 ± 0.01 a	1.98 ± 0.00 b	3.58 ± 0.30 c	4.92 ± 0.14 d	6.45 ± 0.23 e
Cryptochlorogenic acid	0.08 ± 0.00 a	0.16 ± 0.00 b	0.22 ± 0.02 c	0.28 ± 0.00 d	0.34 ± 0.00 e
Caffeoylglycerol	6.26 ± 0.00 b	5.48 ± 0.23 a	5.85 ± 0.00 a	5.80 ± 0.22 a	5.23 ± 0.17 a
p-Coumaryl quinic acid	1.13 ± 0.14 a	0.86 ± 0.20 a	1.17 ± 0.00 a	1.05 ± 0.00 a	1.12 ± 0.11 a
Caffeoyl-dihydroxyphenyl-lactoyl-tartaric acid	2.29 ± 0.10 a	1.93 ± 0.30 a	2.12 ± 0.00 a	2.18 ± 0.00 a	2.46 ± 0.21 a
2-O-p-Coumaroylglycerol	6.28 ± 0.37 c	5.03 ± 0.14 a	5.73 ± 0.00 b	5.16 ± 0.00 a	5.32 ± 0.17 a
1-O-p-Coumaroylglycerol	2.27 ± 0.00 d	2.39 ± 0.12 d	2.21 ± 0.00 c	1.97 ± 0.07 b	1.89 ± 0.00 a
p-Coumaroylspermidine	1.08 ± 0.27 b	1.48 ± 0.00 c	0.80 ± 0.00 a	1.14 ± 0.00 b	0.79 ± 0.00 a
di-p-Coumaroylspermidine	7.82 ± 0.02 c	5.67 ± 0.12 b	5.85 ± 0.21 b	5.02 ± 0.00 a	5.08 ± 0.05 a
Feruloylquinic acid	1.39 ± 0.00 e	1.00 ± 0.00 d	0.99 ± 0.00 c	0.92 ± 0.02 b	0.82 ± 0.00 a
(+) Catechin	0.25 ± 0.02 a	0.38 ± 0.00 b	0.50 ± 0.10 c	1.63 ± 0.00 d	1.47 ± 0.17 d
Procyanidin B2	0.45 ± 0.00 a	0.51 ± 0.00 b	1.16 ± 0.07 c	1.33 ± 0.00 d	1.47 ± 0.24 d
(-) Epicatechin	0.19 ± 0.00 a	0.42 ± 0.00 b	0.68 ± 0.05 c	0.99 ± 0.00 d	1.49 ± 0.13 e
Floretin-2-O-xylo-glucoside	0.01 ± 0.00 a	0.02 ± 0.00 a	0.20 ± 0.01 b	0.29 ± 0.00 c	0.40 ± 0.00 d
Floretin 2-O-glucoside (phloridzin)	0.15 ± 0.00 a	0.87 ± 0.02 b	1.74 ± 0.04 c	3.17 ± 0.00 d	3.71 ± 0.01 e

^∗^For abbreviations, see Table 1. ^∗∗^Different letters in rows denote mean values statistically different from each other.

## Data Availability

The data used to support the findings of this study are included within the article. The raw data will be provided on request.
